# Relationship between Nonalcoholic Fatty Liver Disease and Vitamin D Nutritional Status in Extreme Obesity

**DOI:** 10.1155/2017/9456897

**Published:** 2017-06-08

**Authors:** Adryana Cordeiro, Silvia Pereira, Carlos José Saboya, Andrea Ramalho

**Affiliations:** ^1^Micronutrients Research Center, Federal University of Rio de Janeiro, 21941-590 Rio de Janeiro, RJ, Brazil; ^2^Multidisciplinary Center of Bariatric and Metabolic Surgery, 22280-020 Rio de Janeiro, RJ, 22280-020, Brazil

## Abstract

**Aim:**

To evaluate the relationship of nonalcoholic fatty liver disease (NAFLD) with nutritional status of vitamin D in extreme obesity.

**Methods:**

Descriptive cross-sectional study in individuals with class III obesity (BMI ≥ 40 kg/m^2^), aged ≥ 20 years to < 60 years. Data were obtained for weight, height, waist circumference (WC), and serum 25-hydroxyvitamin D (25(OH)D) levels. Vitamin D analysis was performed by high performance liquid chromatography (HPLC) and the cutoff points used for its classification were < 20 ng/mL for deficiency and 20–29.9 ng/ml for insufficiency. NAFLD gradation was conducted through histological evaluation by liver biopsy.

**Results:**

The sample is comprised of 50 individuals (86% female). BMI and average weight were 44.1 ± 3.8 kg/m^2^ and 121.4 ± 21.4 kg, respectively. Sample distribution according to serum 25(OH)D levels showed 42% of deficiency and 48% of insufficiency. The diagnosis of NAFLD was confirmed in 100% of the individuals, of which 70% had steatosis and 30% had steatohepatitis. The highest percentage of 25(OH)D insufficiency was seen in individuals with steatosis (66%/*n* = 21) and steatohepatitis (93%/*n* = 16). All individuals with steatohepatitis presented VDD (*p* < 0.01).

**Conclusion:**

The results of this study showed high prevalence of serum 25(OH)D inadequacy in individuals with class III obesity, which worsens as the stage of liver disease progresses.

## 1. Introduction

Nonalcoholic fatty liver disease (NAFLD) has become one of the most common chronic liver diseases worldwide [1]. NAFLD is characterized by an accumulation of fat in the liver in the absence of such secondary causes as alcohol abuse, viral hepatitis, and so forth [2], while presenting such wide-ranging histological features as simple macrovesicular steatosis and nonalcoholic steatohepatitis (NASH) that can evolve into fibrosis, cirrhosis, or hepatocellular carcinoma [3].

Vitamin D deficiency (VDD) can result from problems relating to the absorption of vitamin D, hydroxylation due to liver failure, improper dietary intake, or inadequate exposure to sunlight. It is the most prevalent micronutrient deficiency in the world, with a billion people estimated to be deficient [4]. Individuals with obesity, including those suffering from liver disease, are more susceptible to VDD [5]. A potential explanation for this deficiency is, when there is damage of the liver, synthesis of 25(OH)D may be impaired by the presence of steatosis.

VDD can exacerbate NAFLD at least in part through an inflammatory-mediated pathway, given how vitamin D mediates its intracellular signals via the vitamin D receptor (VDR), which is constitutively expressed in the liver [6]. VDR expression in the cholangiocytes and hepatocytes of NAFLD sufferers correlated inversely with the extent of the disease [7].

There is limited information on the potential role VDD plays in NAFLD diagnosed via liver biopsy, mainly where NASH is concerned [8]. Thus, the aim of this study is to investigate the relationship between serum 25(OH)D levels and NAFLD staging, as diagnosed via liver biopsy, in extreme obesity.

## 2. Material and Methods

The study is comprised of 50 individuals with class III obesity (Body mass index [BMI] ≥ 40 Kg/m^2^), of both sexes, aged ≥20 to <60 years, from a clinic specialized in controlling obesity in the municipality of Rio de Janeiro, in the period from January to December 2013. Pregnant women, nursing mothers, patients with malabsorption bowel disorder, acute and chronic infections, and associated endocrinopathies (hypothyroidism and hypocortisolism), subjects with diabetes and on insulin or oral antihyperglycemic agents, individuals who made use of medication or vitamin supplement containing vitamin D, whose alcohol consumption exceeded 20 g/day in women and 40 g/day in men, individuals who used drugs that can increase risk factor for NAFLD (amiodarone, corticosteroids, synthetic estrogen, tamoxifen, and nifedipine), and individuals who had alcoholic liver disease, viral hepatitis, autoimmune hepatitis, primary biliary cirrhosis, primary sclerosing cholangitis, liver metabolic disorders, and drug-induced hepatitis or another liver disease that was not NAFLD were excluded from the study.

### 2.1. Anthropometric Evaluation

Class III obesity classification was based on the World Health Organization (WHO) [9] criteria. BMI calculation was conducted according to anthropometric measurements of weight (kg) and height (m^2^) [9].

Measurement of waist circumference (WC) was performed with the patient standing, relaxed abdomen, arms by the sides, and feet together, and a nonextensible tape measure was used. WC was measured midway between lower rib margin and the superior anterior iliac spine. Measurement was performed at the completion of the individual normal expiration.

### 2.2. Biochemical Evaluation

For biochemical evaluation, blood sample was obtained by venipuncture after a 12 h fasting period. Laboratory tests were conducted to evaluate liver function and damage: albumin, aspartate aminotransferase (AST), alanine aminotransferase (ALT), alkaline phosphatase (AP), gamma-glutamyl transpeptidase (GGT), lipid profile [total cholesterol, triglycerides (TG), high-density lipoprotein cholesterol (HDL-c), low density lipoprotein cholesterol (LDL-c)], glucose, and insulin. Determinations of triglycerides, total cholesterol, HDL-c, and glucose were performed by the enzymatic colorimetric method. Reagents for these biochemical evaluations were purchased from Labtest Diagnóstica S.A., Minas Gerais, Brazil. LDL-C fraction was determined in accordance with the Friedewald's formula. Basal insulin was quantified by reversed-phase high performance liquid chromatography (RP-HPLC), (Labtest Diagnóstica S.A., Minas Gerais, Brazil) and the cutoff point used was 24.9 uUI/mL.

Insulin resistance (IR) was identified by HOMA-IR index obtained from the following calculation: HOMA-IR = fasting insulin (mU/L) × fasting blood glucose (mmol/L/22.5). The reference values used were found in the literature for healthy adult individuals and the cutoff point obtained was above 2.5 [10, 11].

Vitamin D analysis was conducted in the form of 25(OH)D and the method used for its quantification was HPLC [12]. Serum values obtained were compared with the cutoff points for normality according to Endocrine Society clinical practice guideline [13]. Thus, levels of serum concentration of 25(OH)D were classified into deficient (<20 ng/ml), insufficient (20–29.9 ng/ml), and sufficient (≥30 ng/ml and <100 ng/ml). To complete the evaluation of the nutritional status of vitamin D, an investigation was conducted on the sun exposure of the individuals in the study, and the protocol validated by Hanwell and coworkers (2010) [14] was applied.

### 2.3. Diagnosis of NAFLD: Liver Biopsy

Histological evaluation was conducted through a withdrawal of 4 mm thickness of the left lobe of the liver via puncture using a 16 G × 15 cm Menghini needle (Euromed, Minas Gerais, Brazil). Biopsies were conducted by the medical surgeon along with the bariatric surgery. The overall histological evaluations were performed by the same pathologist, who had no knowledge of the biochemical and clinical data of the patients, through staining of parts by hematoxylin-eosin, Masson's trichrome, and Perls' Prussian blue stain (Interlab, São Paulo, Brazil). Hematoxylin-eosin allows a general view of the acinar architecture, inflammatory infiltrates, and changes in hepatocytes. Masson verifies the presence of fibrosis, whether portal, perisinusoidal, or around centrilobular veins. Perls verifies the presence of iron deposits [15].

Grading of NAFLD and staging of hepatic fibrosis were set in accordance with the proposal of Brunt and coworkers [15]. Grading was performed considering the presence of macrovesicular steatosis (simple steatosis) and necroinflammatory activity (presence of NASH).

*Macrovesicular Steatosis*

  Grade 0: no steatosis.
  Grade 1 (mild): <33% of fat accumulation in hepatocytes.
  Grade 2 (moderate): between 33% and 66% of hepatocytes affected.
  Grade 3 (severe): >66% of hepatocytes affected.

*Necroinflammatory Activity (NASH)*

  Grade 0: no steatosis.
  Grade 1 (mild).
  Grade 2 (moderate).
  Grade 3 (severe).

*Staging of Fibrosis Was Performed in Individuals with NASH*

  Stage 0: no steatosis.
  Stage 1: presence of pericellular or perisinusoidal fibrosis in Zone 3, focal or extensive.
  Stage 2: presence of pericellular or perisinusoidal fibrosis in Zone 3 associated with the presence of focal or extensive periportal fibrosis.
  Stage 3: presence of pericellular or perisinusoidal fibrosis in Zone 3 and focal or extensive fibrotic bridges.
  Stage 4: cirrhosis.

### 2.4. Statistical Analysis

Statistical calculations were performed by the SPSS program version 17.0. Statistical analyses used were Student's *t*-test or ANOVA to compare continuous variables expressed as mean and standard deviation (SD); Pearson's correlation; and Spearman's correlation between nonparametric variables; association was verified by the Chi-square (*χ*^2^) test. A 5% significance level was established.

Odds ratio (OR) and 95% CI were calculated using logistic regression to determine the risk of NAFLD for nutritional status of 25(OH)D. Model was controlled for BMI and WC.

This study was approved by the Research Ethics Committee of Hospital Universitário Clementino Fraga Filho of Federal University of Rio de Janeiro (Research Protocol no. 011/06-CEP).

## 3. Results

### 3.1. Description of the Study Population

The sample is comprised of 50 individuals, predominantly for female (*n* = 43/86%), without statistical difference between gender. Characteristics anthropometric and concentration of vitamin D according to gender of the sample were presented in Table 1.

In Table 2, all biochemical variables were shown.

Regarding WC, all patients had values that were above the threshold of 102 cm (men) and 88 cm (women), as set by WHO (1998) [16], for high risk of metabolic complications.

### 3.2. Vitamin D Status in the Subjects of the Study

Prevalence of deficiency and insufficiency of 25(OH)D in the group studied was 42% and 48%, respectively, and prevalence of sufficiency was 10%, showing an inadequacy percentage of 90%. According to 25(OH)D levels stratified by gender, we found a lower mean (17.9 ± 9.8 ng/mL) in men as compared to women, found to have a mean of 22.9 ± 7.2 ng/mL (*p* = 0.165), without significant difference. Prevalence of vitamin D deficiency was higher in males (57.1% versus 39.6%; *p* = 0.04), with virtually no subject meeting the criteria for serum sufficiency of vitamin D according to the cutoff point for normality proposed by Holick and coworkers (2011) [13].

Assessing the time of sun exposure, a mean was 13.2 ± 5.2 minutes using the validated protocol.

### 3.3. Relation between Vitamin D Status and Biochemical Indicators of Liver Function and Damage

Means of AP, ALT, AST, GGT, and albumin did not present significant statistical difference between the groups of nutritional status of vitamin D, as shown in Table 3.

### 3.4. Diagnosis of NAFLD and the Relationship with Serum Concentrations of Biochemical Indicators of Liver Function and Damage

Diagnosis of NAFLD was confirmed in 100% of the patients according to histological evaluation by liver biopsy: 70% of the individuals had steatosis and 30% had steatohepatitis. Among the individuals who had steatohepatitis, 13% presented with fibrosis.

Table 4 shows the mean serum concentrations of biochemical indicators of liver function and damage according to staging of NAFLD. GGT activity was higher in individuals with steatohepatitis (significantly for steatohepatitis with fibrosis versus steatosis); ALT and AST liver enzyme activities were within the normality standard as well as AP levels. Albumin levels were significantly lower in individuals with steatohepatitis with fibrosis.

### 3.5. Diagnosis of NAFLD and the Relationship with Anthropometric Variables and HOMA-IR

Individuals with steatohepatitis with fibrosis had weight, WC, and BMI levels without significant difference when compared with steatosis alone (Table 5). Besides, in these subjects with presence of fibrosis, we noted that HOMA-IR value was higher (5.7 ± 1.2) than individuals with steatosis (4.6 ± 0.9) [*p* = 0.031].

### 3.6. Correlation between Staging of NAFLD with Serum Concentrations of 25(OH)D

When correlating the degree of staging of NAFLD with serum concentrations of 25(OH)D, the current study demonstrated that, in individuals with steatosis, steatohepatitis without fibrosis and steatohepatitis with fibrosis the percentages of sufficiency, insufficiency, and deficiency were 19%, 66%, and 15%; 0%, 18%, and 82%; 0%, 0%, and 100%, respectively. Data showed the highest percentage of deficiency in the group with steatohepatitis with fibrosis.

**Figure 1:**
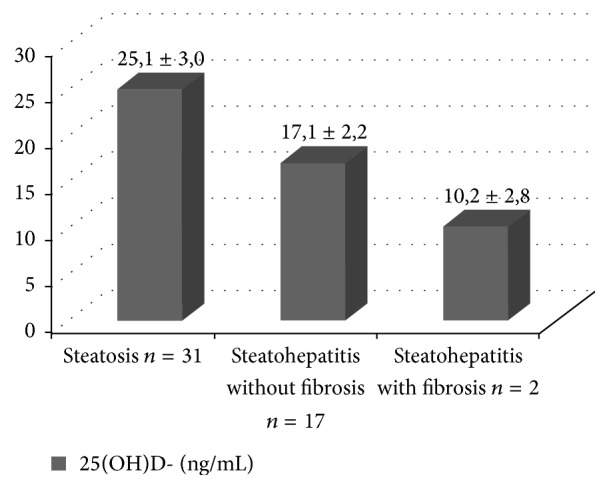
Mean serum concentrations of vitamin D [25(OH)D] by staging of NAFLD by liver biopsy. ANOVA (*p* < 0,01).

A significant difference was observed in the serum concentrations of 25(OH)D when the staging of NAFLD was determined by liver biopsy (*p* < 0.01). The highest mean was found in a milder degree of NAFLD (steatosis) but it showed inadequacy, and the lowest mean of 25(OH)D was found in steatohepatitis with fibrosis, as shown in Figure 1.

### 3.7. Association between Vitamin D and NAFLD by Logistic Regression Analysis

An association between vitamin D and NAFLD according to binary logistic regression analyses was observed. After adjustment for BMI and WC, the OR for NAFLD in the deficiency compared with sufficiency of serum concentrations of vitamin D was 1.92 (95% CI 1.38, 1.98) [*p* = 0.001] and the insufficiency compared with sufficiency was 1.53 (95% CI 1.27, 1.78) [*p* = 0.002].

### 3.8. Correlation between Serum Concentrations of Vitamin D and Anthropometric/Biochemical Parameters

When serum concentrations of vitamin D were considered, a significant negative correlation was found with TG, HOMA-IR, insulin, and WC (*r* = −0.384, *p* < 0.01; *r* = −0.330, *p* < 0.05; *r* = −0.332, *p* = 0.01; *r* = −0.320, *p* = 0.02, resp.).

## 4. Discussion

The results of this study show that the lowest serum vitamin D concentrations in the form of 25(OH)D were found in steatohepatitis diagnosed by liver biopsy, a gold standard method of diagnosis. We demonstrated that VDD is prevalent in patients with NAFLD, mainly in more histologically severe stages of the disease, thus suggesting that vitamin D may play a role in the development and progression of liver disease, partly via inhibition of vitamin D's anti-inflammatory properties [17]. Furthermore, vitamin D's immunomodulatory properties may help explain the impact this vitamin has on the progression and severity of NAFLD [18]. As the liver is a key organ in vitamin D synthesis, by transforming vitamin D_3_ into 25(OH)D, impairment of the organ may explain why vitamin D is found to be inadequate in most liver diseases. A deficit in vitamin D-VDR axis signaling may represent an aggravating factor for these diseases, because VDR expression is low or absent from hepatocytes and the association between vitamin D-VDR axis and pathophysiology may be the result of an alteration in signals from nonparenchymal liver cells or hepatic cells, especially where NAFLD is concerned [19]. Given how low serum 25(OH)D levels are associated with a progression in hepatic fibrosis, the risk of these individuals developing hepatocellular carcinoma increases, since vitamin D acts as an inhibitor of hepatic fibrosis [20] and the expression of detoxifying enzyme in the liver. Additionally, VDR is expressed in hepatic stellate cells, which are major producers of cellular matrix and the main indicators of liver fibrosis [21].

We found that VDD had an independent relationship (OR 1.92; 95% CI 1.38, 1.98) with NAFLD, suggesting that serum 25(OH)D levels protected against NAFLD after adjusting for WC and BMI. VDD could potentiate the metabolic pathways of NAFLD pathogenesis, including immunological, hormonal, and cellular differentiation mechanisms, affecting adipocytokines and proinflammatory cytokines, which are secreted by adipose tissue (AT) and are significant in the progression of NAFLD [22, 23]. Recent studies suggest that VDD is a significant risk factor for the development of NAFLD [24] and indicate that vitamin D's anti-inflammatory and immune modulatory properties may provide plausible mechanisms by which vitamin D may impact NAFLD progression and severity. Moreover, a study from China found that VDD can positively regulate endogenous fatty acid synthesis in NASH via impaired enterohepatic circulation and the administration of 1,25(OH)_2_D_3_ corrected NASH phenotypes in accordance with inflammation and hepatic lipogenesis [25]. In animal studies vitamin D was found to play an important role in the regulation of oxidative stress, the production of proinflammatory cytokines [26], hepatocyte apoptosis, and hepatic fibrosis [27].

In our study, the individuals with higher BMI and body weight were those who suffered steatohepatitis with fibrosis, and further studies highlight how there is a strong association between excessive adiposity and NAFLD and IR. This association causes predisposition to systemic hypertension, dyslipidemia, and inflammation. Additionally, the association between these clinical findings and excess AT involves metabolic and inflammatory mechanisms [28]. Although VDD mechanisms that contribute to the deposition of fat in the liver are not yet well understood, studies show that VDD may be involved in the regulation of insulin action and could relate to a decrease in secretion of this hormone [29], malfunction in the level of insulin reception [30], and the induction of subclinical inflammation [31]. As the association between IR and NAFLD is well-established in the literature, it is reasonable to assume that there is some connection between VDD and liver disease, as reported in the experimental study developed by Roth et al. (2012) [26] in which VDD caused IR due to an increase in the gene expression of liver resistin, inflammatory liver regulation, and genes of oxidative stress. Vitamin D also modulates the metabolism of free fatty acids (FFAs) acting on peroxisome proliferator-activated receptor (PPAR-*γ*), thereby relieving FFA-induced insulin resistance in vitro. Consequently the increased FFAs flowing in the bloodstream could promote fat deposition into the hepatocytes and the progression of NAFLD on condition of VDD [32].

Several studies have shown the relationship between obesity and inadequacy of VD [33–35]; both interact synergistically to influence the risk of insulin resistance [36]. Low serum of 25(OH)D concentrations is found to be inversely correlated with measures of obesity, including BMI (≥30 kg/m^2^), fat mass, and WC [37, 38]. A bidirectional genetic study developed suggested that high BMI led to lower 25(OH)D; each unit increase in BMI is being associated with 1.15% lower concentration of 25(OH)D [39]. In our study we found a negative correlation between WC and serum vitamin D levels, reinforcing findings which demonstrate that more important than the total amount of body fat is its distribution, and the presence of fat in the abdominal region is a predictive factor for the worsening of VDD [40]. In stage of obesity, AT undergoes hypertrophic enlargement which results in an unbalanced blood flow leading to inflammation and macrophage infiltration [41], and as a consequence there is a reduction in adiponectin secretion and an increase in proinflammatory cytokines [42]. Obesity is commonly linked to an upregulation of proinflammatory molecules and downregulation of anti-inflammatory molecules [43]; individuals with both high SAT and high VAT have an approximately threefold prevalence of VDD compared with those with both low SAT and low VAT [44]. 1,25(OH)_2_D_3_ inhibits chronic inflammation in AT by regulating approximately 140 genes favoring oxidative stress and inflammatory biomarkers; reducing IL-6, IL-8, and IL-1*β*; and inhibiting of NF-*κβ* [20, 45–47].

Our study is comprised of a majority of women, which matched the research of Benoit et al. (2014) [48], where 73–80% of the individuals attending a clinic specialized in controlling obesity were females; therefore gender was not confounding factor. A higher prevalence of VDD was found in men, without adequate vitamin D nutritional status, despite the fact that these individuals resided in a city with a great deal of sunlight. Our findings confirm those of Cabral et al. (2013) [49], who reported a high prevalence of vitamin D deficiency in men, similar to those in our study. Some studies report a high prevalence of VDD worldwide [50–52], even in countries at low latitudes, where there is usually higher UVB irradiation, resulting in conditions suitable for synthesis of the vitamin. A recent study involving Brazilian women of reproductive age presented a mean of 25(OH)D as 23.8 ± 8.7 ng/mL [53], values very close to ours. A recent study carried out in the United Kingdom evaluating individuals with class III obesity in preoperative bariatric surgery [54] showed a 90% percentage of serum vitamin D deficiency, assessed in the form of 25(OH)D in the sample studied, data similar to our findings. Among the indicators of hepatocellular damage most widely used in the literature are GGT and AP transaminases [55], but in this study mean levels of ALT, AST, and AP among individuals with hepatic steatosis and steatohepatitis showed no significant differences. Although many studies have reported an increase in indicators of liver function and damage in individuals with various degrees of NAFLD [56, 57], these enzymes generally are normal in over 78% of individuals [58]. Regarding GGT values, our study showed values statistically higher in individuals with steatohepatitis, especially in those with fibrosis, which suggests that the worsening of liver disease may be a possible explanation for increased GGT as liver fibrosis progresses [59].

*Strengths and Weaknesses of the Study*. The present study has some limitations: (1) the number of patients evaluated in the research; (2) the noninclusion of a control group. However, there is still a dearth of literature regarding studies that jointly evaluate the relationship between VDD and NAFLD according to severity of disease. Additionally, our findings highlight the relevance of the NAFLD classification method and staging of hepatic fibrosis through liver biopsy, considering the gold standard for its high diagnostic accuracy.

## 5. Conclusion

The results of this study show high prevalence of serum 25(OH)D inadequacy in individuals with class III obesity, which worsens as the stage of liver disease progresses.

Monitoring vitamin D nutritional status in sufferers of class III obesity and NAFLD is important for the management of their health. Further investigations are needed in order to provide more information regarding new mechanisms in NAFLD physiopathology when associated with VDD.

## Figures and Tables

**Table 1:** Mean of age, characteristics anthropometric, and concentration of vitamin D according to gender of the sample (*n* = 50).

Characteristics	Female (*n* = 43)	Male (*n* = 7)	*p* value
Age (years)	41.3 ± 8.6	45.7 ± 6.6	0.324
BMI (Kg/m^2^)	42.7 ± 3.2	45.8 ± 2.6	0.765
Weight (Kg)	118.6 ± 20.2	124.1 ± 16.5	0.543
WC (cm)	121.6 ± 12.7	119.7 ± 10.1	0.438
Vitamin D-25(OH)D (ng/mL)	22.9 ± 7.2	17.9 ± 9.8	0.165

Mean and standard deviation: BMI: body mass index; WC: waist circumference.

**Table 2:** Biochemical variables of the sample (*n* = 50).

Variables	Mean/SD
Albumin (g/dL)	4.2 ± 0.5
AST (U/L)	25.1 ± 18.3
ALT (U/L)	27.4 ± 15.9
AP (U/L)	76.1 ± 23.4
GGT (U/L)	33.4 ± 21.8
TG (mg/dL)	132.0 ± 50.9
Cholesterol (mg/dL)	197.7 ± 36.1
LDL-c (mg/dL)	121.8 ± 34.5
HDL-c (mg/dL)	48.3 ± 11.86
Insulin (mcU/mL)	19.0 ± 12.5
Glucose (mg/dL)	100.2 ± 19.0
HOMA-IR	4.5 ± 4.7

Mean and standard deviation: ALT: alanine aminotransferase; AP: alkaline phosphatase; AST: aspartate aminotransferase; GGT: gamma-glutamyl transpeptidase; LDL-C: low density lipoprotein cholesterol; HDL-C: high-density lipoprotein cholesterol; HOMA-IR: homeostatic model assessment insulin resistance; TG: triglycerides.

**Table 3:** Mean serum concentrations of biochemical indicators of liver tests in relation to the nutritional status of vitamin D.

Variables	Nutritional status of vitamin D			
	Deficiency	Insufficiency	Sufficiency	*p* value
	*n* = 20	*n* = 24	*n* = 6	
AST (U/L)	28.1 ± 22.8	23.5 ± 14.9	19.4 ± 5.3	0.672
ALT (U/L)	29.1 ± 17.5	26.9 ± 15.8	22.4 ± 9.9	0.587
AP (U/L)	72.7 ± 18.2	81.2 ± 26.1	65.6 ± 28.1	0.234
GGT (U/L)	39.2 ± 25.7	28.9 ± 17.1	30.3 ± 22.8	0.212
Albumin (U/L)	4.4 ± 0.6	4.1 ± 0.3	4.1 ± 0.3	0.287

ANOVA: mean and standard deviation. AST: aspartate aminotransferase; ALT: alanine aminotransferase; AP: alkaline phosphatase; GGT: gamma-glutamyl transpeptidase.

**Table 4:** Mean deviation serum concentrations of biochemical indicators of liver tests according to the staging of NAFLD.

Variables	Staging of NAFLD by liver biopsy			
	Steatosis	Steatohepatitis without fibrosis	Steatohepatitis with fibrosis	*p* value
	(*n* = 31)	(*n* = 17)	(*n* = 2)	
AST (U/L)	22.9 ± 12.8	29.9 ± 26.7	30.2 ± 7.6	0.410
ALT (U/L)	27.1 ± 15.5	28.2 ± 17.5	28.9 ± 10.3	0.343
AP (U/L)	75.1 ± 24.9	78.3 ± 20.2	73.1 ± 1.4	0.722
GGT (U/L)	24.9 ± 17.8	42.6 ± 27.7	62.4 ± 14.6	**0.044**
Albumin (U/L)	4.5 ± 0.7	3.9 ± 0.3	3.3 ± 0.2	**0.029**

ANOVA: mean and standard deviation. Alb: albumin; ALT: alanine aminotransferase; AP: alkaline phosphatase; AST: aspartate aminotransferase; GGT: gamma-glutamyl transpeptidase.

**Table 5:** Mean/deviation of anthropometric variables according to the staging of NAFLD.

Variables	Staging of NAFLD by liver biopsy		
	Steatosis alone	Steatohepatitis with fibrosis	*p* value
BMI (Kg^2^)	43.7 ± 1.9	45.6 ± 2.2	0.447
Weight (Kg)	145.9 ± 36.5	148.5 ± 47.4	0.209
WC (cm)	132.3 ± 11.5	144.0 ± 18.3	0.087

Student's *T-*test*:* mean and standard deviation: BMI: body mass index; WC: waist circumference.
